# Plasma Bile Acid Profile in Patients with and without Type 2 Diabetes

**DOI:** 10.3390/metabo11070453

**Published:** 2021-07-14

**Authors:** Alessandro Mantovani, Andrea Dalbeni, Denise Peserico, Filippo Cattazzo, Michele Bevilacqua, Gian Luca Salvagno, Giuseppe Lippi, Giovanni Targher, Elisa Danese, Cristiano Fava

**Affiliations:** 1 Section of Endocrinology, Diabetes and Metabolism, Department of Medicine, University and Azienda Ospedaliera Universitaria Integrata of Verona, 37126 Verona, Italy; giovanni.targher@univr.it; 2 Section of General Medicine C and Liver Unit, Department of Medicine, University and Azienda Ospedaliera Universitaria Integrata of Verona, 37126 Verona, Italy; andrea.dalbeni@aovr.veneto.it (A.D.); cattazzo.f@gmail.com (F.C.); bevilacqua.michele@yahoo.com (M.B.); cristiano.fava@univr.it (C.F.); 3 Section of Clinical Biochemistry, Department of Neurological, Biomedical and Movement Sciences, University of Verona, 37126 Verona, Italy; denise.peserico@univr.it (D.P.); gianluca.salvagno@univr.it (G.L.S.); giuseppe.lippi@univr.it (G.L.)

**Keywords:** type 2 diabetes, T2DM, bile acids, BA, metabolic syndrome

## Abstract

A paucity of information currently exists on plasma bile acid (BA) profiles in patients with and without type 2 diabetes mellitus (T2DM). We assayed 14 plasma BA species in 224 patients with T2DM and in 102 nondiabetic individuals with metabolic syndrome. Plasma BA levels were measured with ultra-performance liquid chromatography tandem mass spectrometry (UHPLC-MS/MS) technique. Multivariable linear regression analyses were undertaken to assess associations between measured plasma BA species and T2DM status after adjustment for confounding factors. The presence of T2DM was significantly associated with higher plasma concentrations of both primary BAs (adjusted-standardized β coefficient: 0.279, *p* = 0.005) and secondary BAs (standardized β coefficient: 0.508, *p* < 0.001) after adjustment for age, sex, adiposity measures, serum alanine aminotransferase and use of statins or metformin. More specifically, the presence of T2DM was significantly associated with higher levels of plasma taurochenodeoxycholic acid, taurodeoxycholic acid, glycochenodeoxycholic acid, hyodeoxycholic acid, glycodeoxycholic acid, glycolithocholic acid, deoxycholic acid, taurochenodeoxycholic acid, taurodeoxycholic acid, glycochenodeoxycholic acid and glycodeoxycholic acid (adjusted-standardized β coefficients ranging from 0.315 to 0.600; *p* < 0.01 or less), as well as with lower plasma levels of cholic acid (adjusted-standardized β coefficient: −0.250, *p* = 0.013) and taurocholic acid (adjusted-standardized β coefficient: −0.309, *p* = 0.001). This study shows that there are marked differences in plasma BA profiles between patients with and without T2DM. Further research will be needed to better understand how these differences in plasma BA profiles may interplay with the pathophysiology of T2DM.

## 1. Introduction

Bile acids (BAs) are cholesterol catabolites that are mainly synthesized in the liver [1]. In the classic pathway of BA synthesis, cholesterol is hydroxylated in the 7α position by the enzyme CYP7A1 (cytochrome P450 7A1 or also known as cholesterol 7-alpha-monooxygenase) [1]. In the alternative pathway of BA synthesis, cholesterol is first converted to oxysterol prior to being 7α-hydroxylated by the enzymes CYP7B1 or CYP39A1 [1,2]. After these initial steps, several enzymatic reactions occur to generate two primary BAs, i.e., chenodeoxycholic acid (CDCA) and cholic acid (CA) [1,2]. Following hepatic synthesis, BAs are secreted into bile as glycine or taurine conjugates (in a ratio of approximately 3:1 in humans) and play a key role in intestinal lipid absorption, as well as in controlling gut bacteria overgrowth and maintaining intestinal barrier integrity [1,2]. BAs are actively reabsorbed by enterocytes in the terminal ileum to hepatocytes where they are taken up and reused [1]. Although this process is highly efficient, a small proportion of BAs escapes the ileal uptake, is modified by intestinal microbiota and is passively reabsorbed in the colon [1,2]. For such reasons, BAs can be measured in plasma (or serum) at low levels [1]. Gut bacteria metabolize primary BAs to secondary BAs. In the intestine, a portion of conjugated CA and CDCA are de-conjugated by gut bacterial bile salt hydroxylase (BSH) to form deoxycholic acid (DCA) and lithocholic acid (LCA) [2]. In addition, small amounts of CDCA are converted to ursode-oxycholic acid (UDCA) by gut bacterial 7β-hydroxysteroid de-hydrogenase [2]. In humans, the circulating BA pool is highly hydrophobic and mainly consists of CA, CDCA and DCA, which are present in a ratio of nearly 40:40:20 [2].

The presence of BAs in systemic circulation suggests that BAs could directly affect various tissues [1]. Experimentally, it has been demonstrated that BAs can take part in both glucose metabolism and energy regulation, mostly via the activation of farnesoid X receptor (FXR) and the G protein-coupled bile acid receptor 1, also named bile acid membrane-type receptor TGR5 [1,3]. Preclinical studies showed that hepatic insulin resistance and hyperglycemia increase BA synthesis, resulting in alterations in BA composition [1,4]. Animal studies also showed that diabetic (db/db) mice have a larger total BA pool size than wild type control animals [5].

Presently, it is uncertain whether these alterations in plasma BA profiles may also be detectable in people with type 2 diabetes mellitus (T2DM). The human studies available so far [6,7,8,9,10,11] have yielded inconsistent findings. In some studies, for instance, serum or plasma fasting levels of total BAs were found to be similar between subjects with and without T2DM [6,7,9,11], whereas in other studies only few BA fractions were found to be higher in patients with T2DM than in nondiabetic controls [8,10].

Since BAs are relevant signaling molecules in both glucose and energy homeostasis and some BA sequestrants (e.g., colesevelam) may improve glycaemic control in patients with T2DM [10,12], we reasoned that further investigation of plasma BA profiles in people with T2DM might be useful for improving our current knowledge on the potential role of plasma BA levels in the pathophysiology of T2DM. Therefore, in this cross-sectional study, we assessed whether patients with established T2DM had some differences in plasma BA levels compared to subjects without T2DM and whether the presence of T2DM was associated with plasma BA levels independent of potential confounding variables.

## 2. Results

Main clinical and biochemical characteristics of participants, stratified by T2DM status, are reported in Table 1. Compared with those without T2DM, patients with T2DM were more likely to be older, were men and were centrally overweight or obese and had higher serum triglyceride and glucose concentrations. Patients with T2DM also had higher proportions of hypertension, dyslipidemia, prior history of IHD, VHD, permanent AF and were more likely to be treated with statins, anti-platelet or anti-hypertensive drugs. In contrast, patients with T2DM had lower values of diastolic blood pressure, serum total cholesterol, LDL-cholesterol and liver enzymes than those without T2DM. Smoking history and circulating levels of HDL-cholesterol, CRP, creatinine or eGFR_CKD-EPI_ did not significantly differ between the two groups. As reported in Table 1, most of patients with T2DM were treated with metformin (78.6%) followed by sulphonylureas (28.6%), DPP-4 inhibitors (23.7%), GLP-1 receptor agonists (18.3%), SGLT-2 inhibitors (9.8%) or pioglitazone (8.5%). Moreover, 84 (37.5%) patients with T2DM were treated with two glucose-lowering agents, whereas 45 (20.1%) patients were treated with three glucose-lowering agents. By study design, none of our patients with T2DM were treated with insulin.

Table 2 shows plasma BA levels in participants stratified by T2DM status. Patients with T2DM had significantly higher plasma levels of total BAs, as well as higher plasma levels of both primary and secondary BAs. In particular, plasma levels of TCDCA, TDCA, GCDCA, GDCA, GLCA and DCA were significantly higher in patients with T2DM than in those without T2DM. Conversely, plasma levels of CA and TCA were lower in patients with T2DM than in their counterparts without diabetes. No significant differences were found in plasma levels of TUDCA, GUDCA, GCA, UDCA, HDCA and GLCA between the two groups of individuals.

Plasma levels of BAs in the total population, which are simultaneously stratified by sex and T2DM status, are reported in Supplementary Table S1. Among men, patients with T2DM had significantly lower plasma TCA levels and higher plasma levels of TCDCA, TDCA, GCDCA, HDCA, GDCA, GLCA and DCA than compared with those without T2DM. Among women, patients with T2DM had higher plasma levels of TCDCA, TDCA, GCDCA, HDCA, GDCA, GLCA and DCA, but possessed lower levels of CA and TCA than compared with those without T2DM.

Table 3 shows the plasma BA levels in the total population, which are simultaneously stratified by T2DM status and statin use. In particular, T2DM patients who were not treated with statins had significantly higher plasma levels of GUDCA, GCA, TCDCA, GCDCA, HDCA, GDCA, CDCA, GLCA and DCA when compared with both T2DM patients treated with statins and non-diabetic subjects, regardless of the use of statins. In addition, the former also had higher plasma levels of total BA as well as higher levels of both primary and secondary BAs. These differences in BA levels remained statistically significant even after adjustment for age, sex and BMI (by using analysis of covariance). The inter-group comparisons also showed that T2DM patients, irrespective of statin use, had significantly different levels of plasma TUDCA, GUDCA, GCA, UDCA, CA, GCDCA and CDCA, as well as different levels of plasma total and primary or secondary BAs than compared with non-diabetic subjects.

Plasma levels of BAs in the total population, simultaneously stratified by T2DM status and use of metformin are reported in Supplementary Table S2. Specifically, T2DM patients treated with metformin had significantly higher levels of TCDCA, TDCA, HDCA, GDCA, GLCA and DCA when compared with both non-diabetic subjects and T2DM patients who were not treated with metformin. T2DM patients treated with metformin had also significantly lower levels of CA and TCA than compared to the other groups. These significant differences remained essentially unchanged even after adjustment for age, sex and BMI. The inter-group comparisons also showed that T2DM patients, irrespective of metformin use, had significantly different levels of plasma GCA, TCDCA, CA, HDCA, GDCA, CDCA, DCA and TCA.

Supplementary Table S3 shows plasma levels of BAs in the total population, which are simultaneously stratified by T2DM status and use of incretins (i.e., DPP-4 inhibitors or GLP-1 receptor agonists). In particular, T2DM patients treated with incretins had significantly higher plasma levels of TCDCA, GDCA, GLCA and DCA when compared to both non-diabetic subjects and T2DM patients that were not treated with incretins. T2DM patients treated with incretins also had lower plasma levels of TCA. These differences in the aforementioned plasma BA levels remained significant even after adjustments for age, sex and BMI. The inter-group comparisons also revealed that T2DM patients, irrespective of incretin use, had significantly different levels of plasma CDCA and DCA.

Supplementary Table S4 shows the results of a correlation matrix among each plasma BA level, fasting glucose level and plasma lipid profile. Most of measured BAs were highly correlated to each other. Significant correlations were also observed between total cholesterol (or LDL-cholesterol) and some BAs such as TCDCA, TDCA, CA, GCDCA, HDCA, GDCA, GLCA or TCA. Plasma triglycerides were significantly correlated only with UDCA. Finally, significant correlations were also found between fasting glucose levels with TCDCA, TDCA, CA, GCDCA, HDCA, GDCA, GLCA, DCA or TCA.

Table 4 and Table 5 show the associations of primary and secondary BA levels, as well as the associations of each individual BA level with T2DM status with or without coexisting use of metformin, respectively. As shown in Table 4, after adjusting for age, sex, BMI, serum ALT levels and statin use, the presence of T2DM, irrespective of metformin use, was significantly associated with higher levels of both total primary and secondary BAs. As shown in Table 5, after adjusting for age, sex, BMI, serum ALT levels and statin use, the presence of T2DM, irrespective of metformin use, was significantly associated with higher plasma levels of TCDCA, TDCA, GCDCA and GDCA. However, the use of metformin was also associated with higher plasma levels of HDCA, GLCA and DCA as well as with lower plasma levels of CA and TCA. Notably, these results remained essentially unchanged even after adjustment for multiple comparisons by using the Bonferroni’s method. Almost similar results were observed when we additionally adjusted for hypertension or when we adjusted for waist circumference instead of BMI (data not shown).

## 3. Discussion

The main findings of our cross-sectional study are as follows: (1) Ambulatory patients with T2DM had significantly higher plasma levels of primary and secondary BAs compared with nondiabetic control individuals; (2) in particular, patients with T2DM had significantly higher plasma levels of TCDCA, TDCA, HDCA, GDCA, GLCA and DCA, but lower plasma CA and TCA levels; and (3) in multivariable regression analyses, the presence of T2DM (with or without coexisting use of metformin) was one of the strongest predictors of plasma BA levels even after adjusting for age, sex, adiposity measures, serum ALT levels and statin use.

To date, whilst there is convincing experimental evidence that diabetic (db/db) mice have a larger total BA pool size than wild type control animals (4), it is still uncertain whether alterations in circulating BA levels are also present in patients with T2DM. Indeed, the currently available human studies have provided conflicting results, with some suggesting that plasma (or serum) fasting levels of total BAs are similar between individuals with and those without T2DM [6,7,9] and others suggesting that only specific BA fractions are higher in people with T2DM than in nondiabetic control individuals [8,10,11]. For instance, in a small cross-sectional study including 62 subjects with normal glucose tolerance, 25 subjects with impaired glucose tolerance and 12 patients with T2DM, Wewalka et al. reported that fasting taurine-conjugated BA concentrations (especially TUDCA, TCA, TCDCA and TDCA) were higher in patients with T2DM than in the other groups of individuals [10]. In another observational study involving 1707 Chinese patients with T2DM and 1707 control subjects matched for age, sex, BMI and fasting glucose levels, Liu et al. showed that higher levels of conjugated primary BAs (especially TCA, GCDCA, TCDCA and GCDCA) and secondary BA (mainly TUDCA) were associated with an increased risk of T2DM [11]. Conversely, in a cross-sectional study including 71 South Korean drug-naïve patients with T2DM and 95 subjects with impaired fasting glucose and 75 healthy controls, Lee et al. reported that plasma BA profiles were essentially superimposable among these three groups of individuals [7].

Collectively, our findings confirm and expand these previous observations by showing that plasma levels of primary BAs and secondary BAs (especially TCDCA, TDCA, HDCA, GDCA, GLCA and DCA—most of which are conjugated BAs) were significantly higher in patients with T2DM (treated or not with metformin) than in subjects without T2DM, even after adjusting for age, sex, adiposity measures, serum ALT levels, hypertension and use of statins (that are able to modulate plasma BA levels by several mechanisms [13]). These findings support the hypothesis that patients with T2DM have increased transition from unconjugated BAs to conjugated BAs, possibly owing to alterations in activity of multiple enzymes involved in the synthesis and metabolism of BAs. This hypothesis might also be indirectly supported by the fact that, in our study, the calculated ratios between some conjugated and unconjugated BAs were significantly higher in patients with T2DM than in those without (e.g., GCA+TCA/CA ratio: 9.7 ± 14.9 vs. 6.5 ± 14.5; and GDCA+TDCA/DCA ratio: 1.7 ± 2.6 vs. 0.8 ± 0.7, respectively, *p* = 0.001 by the Mann–Whitney test). The conjugation of unconjugated BAs to glycine or taurine is mainly catalyzed by bile acid CoA:amino acid N-acyltransferase (BAAT) and bile acid-Co-A synthase (BACS) [10]. Evidence from the European Prospective Investigation into Cancer and Nutrition (EPIC) study also suggested that specific genetic variants in these enzymes may play a role in T2DM development [14]. The study by Wewalka et al. also provided some evidence on the potential role of BAAT and BACS in maintaining glucose homeostasis [10]. Another possible explanation for the differences in plasma BA profiles we observed between patients with and those without T2DM might be due to presence of altered intestinal barrier permeability (thus contributing to increase the permeability to various luminal factors, including BAs), which has been experimentally documented in animal models of diabetes [15]. Interestingly, in our study, we also observed a different BA profile between T2DM patients treated with or without metformin. Experimental studies suggested that metformin may alter gut microbiota composition as well as the BSH activity in patients with T2DM, thereby increasing some BAs that may antagonize intestinal FXR [2,16]. Conversely, in our study, we found that the impact of incretins (i.e., DPP-4 inhibitors and GLP-1 receptor agonists) on plasma BAs concentrations was modest. Further research is required to better decipher the role of BA-related processes in T2DM pathogenesis and the differential impact of some glucose-lowering drugs on plasma BA profiles.

Unlike some previous Asian studies [7,11], we observed that plasma concentrations of DCA (which is a secondary BA) were significantly higher in patients with T2DM (especially in those treated with metformin) than in those without T2DM. This difference might be due, at least in part, to differences in sample size and subject characteristics, including ethnicity-related variations in genetic factors, body composition, lifestyle habits and pharmacological therapies. Similar to the study by Liu et al. [11], we reported that plasma levels of both CA (i.e., a primary BA) and TCA (which is the taurine-conjugated CA) were lower in patients with T2DM than in those without T2DM. In this regard, it is important to note that CA seems also to possess some anti-diabetic effects, possibly by increasing insulin secretion [11,17] and, hence, its plasma concentrations might be altered in patients with T2DM. The specific role of TCA on glucose metabolism is poorly understood to date, although it seems that, under specific conditions, TCA may be converted to DCA, which activates intestinal FXR and TGR5 signaling pathways to modulate glucose metabolism [2].

Collectively, we believe that the findings of our study might have some important research implications. In particular, since our patients with T2DM had significantly different plasma BA profiles compared to nondiabetic individuals, these results further reinforce the importance of better understanding the differential effects of unconjugated and conjugated BAs on glucose metabolism as well as the possible impact of BA sequestrants for improving glycaemic control in patients with T2DM.

Our study has some important limitations that should be mentioned. Firstly, the cross-sectional design of the study does not allow us to draw causal and temporal inferences about the observed associations. Secondly, detailed information regarding dietary habits, insulin resistance, steroid metabolism and oxidative stress was not available. Thirdly, gut microbiota composition (which is mainly responsible for the production of secondary BAs) was not assessed in our study. Fourthly, we did not measure urinary BA excretion. Finally, although in our multivariable regression models we have performed adjustment for multiple potential confounders, residual confounding cannot be definitely excluded.

Notwithstanding these limitations, our study also possesses important strengths, such as the relatively large sample size; the exclusion of patients with known cirrhosis, chronic liver diseases, cancer or end-stage renal disease (we believe that the inclusion of such patients would have confounded the interpretation of data); the completeness of the dataset and plasma BA profiles (the profiles were measured using a validated ultra-high performance liquid chromatography tandem mass spectrometry [UHPLC-MS/MS]); and the ability to adjust for multiple risk factors and potential confounders (including the use of some drugs that may influence the levels of plasma BAs, such as metformin and statins).

In conclusion, the results of our cross-sectional study show that that both primary and secondary plasma BA concentrations (especially TCDCA, TDCA, HDCA, GDCA, GLCA and DCA levels) were significantly higher in patients with T2DM than in individuals without T2DM. Conversely, plasma CA and TCA concentrations were lower in T2DM patients. These associations between each specific BA and T2DM remained statistically significant even after adjusting for age, sex, adiposity measures, serum ALT levels, hypertension and use of statins and metformin. However, further studies will be needed for corroborating these findings in other independent cohorts and for better elucidating the existing but complex links between plasma BA profiles and T2DM.

## 4. Materials and Methods

### 4.1. Participants

We used two different historical cohorts of individuals: (a) patients with established T2DM (*n* = 224) who regularly attended the Diabetes outpatient service at the University Hospital of Verona over a period of 8 months (most of these T2DM patients (*n* = 157) have been included in a previous study) [18]; and (b) nondiabetic patients (*n* = 102) with established metabolic syndrome who regularly attended the Metabolic Disease Clinic and Liver Clinic at the University Hospital of Verona over a period of 10 months.

The following were excluded from the present study: (a) patients with cirrhosis of any aetiology, chronic liver diseases, active cancers or end-stage kidney disease (defined as estimated glomerular filtration rate <15 mL/min/1.73 m^2^ or chronic dialysis); (b) those with documented history of overt hyperthyroidism or hypothyroidism; (c) those who were chronically treated with steroids or estrogen-progestin drugs (for women); and (d) patients with T2DM who were treated with insulin.

The local Ethics Committee approved the study protocol (protocol number: 2004CESC and 2089CESC). All participants gave their written informed consent for participation in this research.

### 4.2. Clinical and Laboratory Variables

Body mass index (BMI) was measured as kilograms divided by the square of height in meters. Waist circumference was measured at the midpoint between the lowest rib and the iliac crest. Blood pressure was measured with a standard sphygmomanometer after the subject had been seated quietly for at least 5 min. Subjects were considered to have hypertension if their blood pressure was ≥140/90 mmHg or if they were taking any anti-hypertensive drugs. Patients were considered to have dyslipidemia if their total cholesterol level was >5.2 mmol/L (>200 mg/dL) or if they were taking statins or other lipid-lowering agents. Metabolic syndrome was diagnosed by using the Adult Treatment Panel (ATP)-III definition [19,20]. In accordance with this definition, a subject was classified as having the metabolic syndrome if he/she had at least three of the following metabolic risk abnormalities: (i) waist circumference >102 cm in men or >88 in women; (ii) triglycerides ≥150 mg/dL; (iii) HDL-cholesterol <40 mg/dL in men and <50 mg/dL in women or those receiving any lipid-lowering drugs; (iv) blood pressure ≥130/85 mmHg or those receiving any anti-hypertensive drugs; and (v) fasting glucose ≥100 mg/dL [19,20]. Information on smoking history and information on the current use of medications were obtained from all participants via interviews during medical examinations.

Venous blood samples were collected in the morning after an overnight fast. Measurements of serum glucose, lipids, creatinine (measured using a Jaffè rate blanked and compensated assay), alanine aminotransferase (ALT), gamma-glutamyltransferase (GGT), C-reactive protein (CRP) and other biochemical blood parameters were obtained using standard laboratory procedures at the central laboratory of our hospital. For patients with T2DM, hemoglobin A1c (HbA1c) levels were measured using the high-performance liquid chromatography analyzer Tosoh-G7 (Tosoh Bioscience Inc., Tokyo, Japan). The estimated glomerular filtration rate (eGFR) was estimated using the Chronic Kidney Disease Epidemiology Collaboration (CKD-EPI) equation [21]. Presence of ischemic heart disease (IHD) was defined as a documented history of myocardial infarction, angina or coronary revascularization procedures. Presence of valvular heart disease (VHD) was defined as described in the medical records, including echocardiogram results. The presence of permanent atrial fibrillation (AF) was defined as described in the medical records, including standard 12 lead electrocardiograms. For patients with T2DM, information on diabetic retinopathy was also recorded.

### 4.3. Plasma BA Measurements

Plasma BA levels were measured using a validated ultra-high performance liquid chromatography tandem mass spectrometry (UHPLC-MS/MS) technique, as described elsewhere [22,23]. Briefly, separation and quantification of individual BAs were performed on Acquity UPLC I-Class System FTN coupled with a Xevo TQ-S micro MS/MS detector (Waters Corporation, Milford, MA, USA) operating in multiple reaction monitoring (MRM) and electrospray negative ionisation mode (ESI−). In all study participants, we assayed the following 14 plasma BAs: tauroursodeoxycholic acid (TUDCA), taurocholic acid (TCA), glycoursodeoxycholic acid (GUDCA), glycocholic acid (GCA), taurochenodeoxycholic acid (TCDCA), taurodeoxycholic acid (TDCA), cholic acid (CA), ursodeoxycholic acid (UDCA), glycochenodeoxycholic acid (GCDCA), hyodeoxycholic acid (HDCA), glycodeoxycholic acid (GDCA), chenodeoxycholic acid (CDCA), glycolithocholic acid (GLCA) and deoxycholic acid (DCA) levels. Among these measured BAs, primary BAs included CA, CDCA and their glycine-conjugates and taurine-conjugates, such as GCA, GCDCA, TCA and TCDCA, whereas secondary BAs (which are generated by deconjugation and/or dehydroxylation of primary BAs by intestinal bacteria) included DCA, UDCA, HDCA and their glycine-conjugates and taurine-conjugates, such as GDCA, TDCA, GUDCA, TUDCA and GLCA. Six internal standards were used: taurocholic acid-d4 (d4-TCA), glycocholic acid-d4 (d4-GCA), cholic acid-d4 (d4-CA), ursodeoxycholic acid-d4 (d4-UDCA), chenodeoxycholic acid-d4 (d4-CDCA) and deoxycholic acid-d4 (d4-DCA). An eight-point calibration curve was used, starting from methanolic standards, with linearity between 5 and 5000 ng/mL. Instrument data were collected and analyzed using MassLynx V4.2 SCN977 (Waters Corporation, Milford, MA, USA). Plasma BA concentrations lower than the lower limit of quantitation (5 ng/mL for each plasma BA) were imputed as 5/sqrt(2) ng/mL [23,24].

### 4.4. Statistical Analysis

Data are expressed as means ± standard deviation (SD) or medians and range inter-quartiles (IQRs) or percentages. Differences between subjects with and without T2DM were tested by the chi-squared test for categorical variables, the Student *t*-test for normally distributed continuous variables, the Mann–Whitney test for non-normally distributed variables (i.e., serum triglycerides, liver enzymes, CRP, eGFR_CKD-EPI_ as well as all measured BA species) and the Dunn’s post-hoc test for the inter-group differences. A multivariable linear regression analysis was used to test the independent association between each plasma BA (logarithmically transformed before statistical analyses and then included as the dependent variable in each regression model) and T2DM status with or without the use of metformin (i.e., non-diabetic subjects vs. T2DM patients not treated with metformin vs. T2DM patients treated with metformin), after adjusting for potential confounding factors. In particular, we performed forced-entry linear regression models adjusted for age, sex, BMI, serum ALT levels and the use of statins (adjusted model 1). In these regression models, we also performed a multiple testing correction using the Bonferroni’s method (i.e., with a *p*-value for significance that was set at 0.05/14 measured BAs = 0.0036) [25]. Similar multivariable linear regression models were also performed to test the independent association between total Bas, primary or secondary plasma BA levels (logarithmically transformed before statistical analyses and then included as the dependent variable in each regression model) and T2DM status with or without the coexisting use of metformin, after adjusting for the same list of the aforementioned covariates. Covariates included in these multivariable regression models were selected as potential confounding factors based on their significance in univariable analyses or based on their biological plausibility. A *p*-value < 0.05 was considered statistically significant. All statistical analyses were performed using the STATA^®^ software, version 16.1 (Stata Corporation, College Station, TX, USA).

## Figures and Tables

**Table 1:** Main clinical and biochemical characteristics of patients stratified by presence/absence of type 2 diabetes mellitus (T2DM).

	Without T2DM (*n* = 102)	With T2DM (*n* = 224)	*p*-Values
Age (years)	51 ± 10	69 ± 10	<0.001
Male sex (%)	21.6	53.6	<0.001
Current smokers (%)	14.0	15.0	0.813
BMI (kg/m^2^)	26.9 ± 4.1	28.7 ± 4.7	<0.001
Waist circumference (cm)	98 ± 13	101 ± 13	0.028
Systolic blood pressure (mmHg)	132 ± 12	134 ± 18	0.250
Diastolic blood pressure (mmHg)	82 ± 8	76 ± 10	<0.001
Fasting glucose (mg/dL)	92 ± 12	128 ± 29	<0.001
HbA1c (%) *	not measured	7.0 ± 0.8	NA
Total cholesterol (mmol/L)	5.0 ± 0.8	4.0 ± 0.9	<0.001
LDL-cholesterol (mmol/L)	3.2 ± 0.7	2.0 ± 0.8	<0.001
HDL-cholesterol (mmol/L)	1.4 ± 0.4	1.4 ± 0.4	0.928
Triglycerides (mmol/L)	1.0 (0.8–1.5)	1.2 (0.9–1.7)	<0.001
ALT (IU/L)	27 (17–39)	13 (10–17)	<0.001
GGT (IU/L)	25 (18–48)	19 (14–29)	<0.001
CRP (mg/L)	1.0 (1.0–2.2)	1.2 (0.6–2.9)	0.059
Creatinine (umol/L)	77 ± 13	79 ± 30	0.423
eGFR_CKD-EPI_ (mL/min/1.73 m^2^)	80 (71–96)	81 (67–93)	0.161
Hypertension (%)	63.7	82.0	<0.001
Dyslipidemia (%)	46.1	83.5	<0.001
Prior IHD (%)	0	16.6	<0.001
Prior VHD (%)	0	20.1	<0.001
Permanent AF (%)	0	3.6	0.034
Diabetic retinopathy (any degree) (%)	NA	14.0	NA
Beta-blocker users (%)	14.7	33.9	<0.001
Ca-channel antagonist users (%)	39.2	23.5	0.003
Diuretic users (%)	11.8	33.9	<0.001
ACE inhibitors/ARB users (%)	46.1	64.6	0.001
Anti-platelet users (%)	0	50.2	<0.001
Statin users (%)	10.8	79.3	<0.001
Metformin users (%) *	NA	78.6	NA
Sulphonylurea users (%) *	NA	28.6	NA
Pioglitazone users (%) *	NA	8.5	NA
GLP-1 receptor agonist users (%) *	NA	18.3	NA
SGLT-2 inhibitor users (%) *	NA	9.8	NA
DPP-4 inhibitor users (%) *	NA	23.7	NA

Sample size, *n* = 326. Data are expressed as means ±SD, medians and interquartile ranges (in parenthesis) or percentages. Differences between the two groups were tested by the chi-squared test for categorical variables, the Student *t*-test for normally distributed continuous variables and the Mann–Whitney test for non-normally distributed variables (i.e., serum triglycerides, ALT, GGT, CRP and eGFR_CKD-EPI_). * Information or data available only in patients with established T2DM (*n* = 224). Abbreviations: ACE: angiotensin-converting enzyme; AF: atrial fibrillation; ALT: alanine aminotransferase; AST: aspartate aminotransferase; ARBs: angiotensin II receptor blockers; BMI: body mass index; CRP: C-reactive protein; DPP-4: dipeptidyl peptidase-4; eGFR: estimated glomerular filtration rate; GGT: gamma-glutamyltransferase; GLP-1: glucagon-like peptide-1; IHD, ischemic heart disease; NA: not applicable; SGLT-2: sodium/glucose cotransporter-2; VHD: valvular heart disease. NB: dyslipidemia was defined as total cholesterol >5.2 mmol/L and/or statin use. Hypertension was defined as blood pressure ≥140/90 mmHg and/or use of any anti-hypertensive agents.

**Table 2:** Plasma bile acid concentrations in patients stratified by presence or absence of type 2 diabetes (T2DM).

	Without T2DM (*n* = 102)	With T2DM (*n* = 224)	*p*-Values
Individual BAs			
TUDCA (ng/mL)	3.5 (3.5–3.5)	3.5 (3.5–3.5)	0.582
GUDCA (ng/mL)	32.1 (13.7–57.7)	22.8 (11.4–56.8)	0.143
GCA (ng/mL)	40.4 (25.6–77.1)	45.6 (23.7–83.8)	0.688
TCDCA (ng/mL)	13.7 (7.9–30.9)	41.6 (21.8–71.3)	<0.001 *
TDCA (ng/mL)	3.5 (3.5–10.4)	12.8 (3.5–31.0)	<0.001 *
UDCA (ng/mL)	11.5 (3.5–27.4)	7.7 (3.5–26.2)	0.564
CA (ng/mL)	19.7 (8.6–72.4)	7.6 (3.5–26.2)	<0.001 *
GCDCA (ng/mL)	109.1 (58.6–191.4)	253.2 (132.4–492.5)	<0.001 *
HDCA (ng/mL)	3.5 (3.5–3.5)	3.7 (3.5–5.5)	<0.001 *
GDCA (ng/mL)	31.3 (17.3–75.8)	97.4 (54.4–189.9)	<0.001 *
CDCA (ng/mL)	51.7 (23.4–131.7)	48.7 (14.7–135.1)	0.297
GLCA (ng/mL)	3.5 (3.5–3.5)	3.7 (3.5–6.5)	<0.001 *
DCA (ng/mL)	93.1 (38.2–171.7)	127.7 (66.4–245.6)	0.002 *
TCA (ng/mL)	17.7 (10.8–32.1)	7.0 (5.0–15.8)	<0.001 *
Total BAs			
Total BAs (ng/mL)	575.2 (361.0–1072.3)	930.2 (557.2–1457.8)	<0.001
Total primary BAs (ng/mL)	318.8 (168.8–731.4)	466.3 (268.8–965.5)	0.002
Total secondary BAs (ng/mL)	213.9 (124.9–388.3)	339.3 (204.6–605.1)	<0.001

Sample size, *n* = 326. Data are expressed as medians and interquartile ranges (in parenthesis). Differences between the two patient groups were tested by the Mann–Whitney test. * These associations remained statistically significant even after adjustment for multiplicity by using the Bonferroni’s correction method (*p*-value was set at 0.05/14 measured BAs = 0.0036). Abbreviations: TUDCA, Tauroursodeoxycholic acid; TCA, taurocholic acid; GUDCA, glycoursodeoxycholic acid; GCA, glycocholic acid; TCDCA, taurochenodeoxycholic acid; TDCA, taurodeoxycholic acid; CA, cholic acid; UDCA, ursodeoxycholic acid, GCDCA, glycochenodeoxycholic acid; GDCA, glycodeoxycholic acid; CDCA, chenodeoxycholic acid; GLCA, glycolithocholic acid; DCA, deoxycholic acid; HDCA, hyodeoxycholic acid.

**Table 3:** Plasma BA concentrations in the whole population simultaneously stratified by T2DM status and statin use.

	Without T2DM and without Use of Statins (*n* = 91) (Group A)	Without T2DM and with Use of Statins (*n* = 11) (Group B)	With T2DM and without Use of Statins (*n* = 46) (Group C)	With T2DM and with Use of Statins (*n* = 178) (Group D)	*p*-Values for Trend	*p*-Values for Trend *	*p*-Values for A vs. B	*p*-Values for A vs. C	*p*-Values for A vs. D	*p*-Values for B vs. C	*p*-Values for B vs. D	*p*-Values for C vs. D
Individual BAs												
TUDCA (ng/mL)	3.5 (3.5–3.5)	3.5 (3.5–3.5)	3.5 (3.5–3.5)	3.5 (3.5–3.5)	0.471	0.351	0.263	0.001	0.231	0.009	0.365	<0.001
GUDCA (ng/mL)	32.4 (12.8–57.1)	20.2 (14.9–102.4)	49.9 (15.4–83.1)	21.6 (10.2–49.4)	0.010	0.006	0.447	0.097	0.018	0.283	0.156	0.001
GCA (ng/mL)	43.1 (24.9–78.8)	31.5 (27.5–42.8)	66.4 (45.2–140.5)	38.7 (22.1–71.9)	<0.001	<0.001	0.123	0.002	0.172	0.004	0.213	<0.001
TCDCA (ng/mL)	15.1 (7.9–31.4)	8.8 (6.4–11.9)	46.4 (26.0–90.8)	21.6 (39.9–70.8)	<0.001	<0.001	0.069	<0.001	<0.001	<0.001	<0.001	0.109
TDCA (ng/mL)	3.5 (3.5–10.7)	3.5 (3.5–9.2)	17.1 (3.5–41.6)	12.5 (3.5–28.1)	<0.001	0.002	0.212	<0.001	<0.001	0.002	0.004	0.177
UDCA (ng/mL)	10.9 (3.5–26.7)	14.1 (3.5–27.9)	12.3 (3.5–46.7)	7.2 (3.5–23.8)	0.289	0.343	0.423	0.161	0.160	0.363	0.273	0.033
CA (ng/mL)	19.6 (8.0–71.3)	36.3 (17.1–106.9)	28.4 (6.4–127.8)	7.2 (3.5–33.9)	<0.001	<0.001	0.170	0.376	<0.001	0.141	0.002	0.001
GCDCA (ng/mL)	111.4 (56.3–200.8)	95.7 (61.1–168.3)	411.5 (211.6–752.2)	222.2 (120.2–387.2)	<0.001	<0.001	0.171	<0.001	<0.001	<0.001	0.002	<0.001
HDCA (ng/mL)	3.5 (3.5–3.5)	3.5 (3.5–3.5)	3.5 (3.5–5.3)	3.5 (3.5–5.5)	0.001	0.007	0.500	<0.001	<0.001	0.017	0.004	0.272
GDCA (ng/mL)	31.7 (17.8–78.7)	23.6 (15.0–72.3)	153.9 (73.8–305.8)	91.4 (53.3–160.6)	<0.001	<0.001	0.330	<0.001	<0.001	<0.001	0.001	0.015
CDCA (ng/mL)	49.8 (23.5–140.1)	54.6 (22.9–110.5)	82.9 (29.3–243.1)	42.7 (13.2–112.0)	0.049	0.046	0.384	0.126	0.043	0.184	0.339	0.005
GLCA (ng/mL)	3.5 (3.5–3.5)	3.5 (3.5–3.5)	3.5 (3.5–9.4)	3.5 (3.5–5.8)	0.008	0.039	0.172	0.001	<0.001	0.005	0.008	0.260
DCA (ng/mL)	99.5 (45.0–172.1)	49.8 (22.1–141.4)	163.8 (82.3–303.7)	124.8 (64.2–243.6)	0.011	0.076	0.200	0.005	0.009	0.015	0.032	0.171
TCA (ng/mL)	18.6 (11.1–35.1)	11.5 (8.9–17.0)	8.5 (5.8–16.8)	7.0 (5.0–14.8)	<0.001	<0.001	0.089	<0.001	<0.001	0.143	0.029	0.081
Total BAs												
Total BAs (ng/mL)	573.7 (361.3–1106.5)	576.6 (343.0–842.6)	1374.8 (836.0–2655.6)	786.6 (517.5–1262.5)	<0.001	<0.001	0.269	<0.001	0.004	<0.001	0.042	<0.001
Total primary BAs (ng/mL)	327.0 (182.1–737.1)	320.5 (157.8–518.4)	918.2 (386.8–1555.7)	429.0 (249.6–781.3)	<0.001	<0.001	0.271	<0.001	0.047	<0.001	0.093	<0.001
Total secondary BAs (ng/mL)	231.6 (125.7–391.6)	201.5 (90.3–215.4)	515.1 (272.9–859.9)	320.7 (194.2–539.3)	<0.001	<0.001	0.228	<0.001	<0.001	<0.001	0.014	0.003

Sample size, *n* = 326. Data are expressed as medians and interquartile ranges (in parenthesis). Differences among the four patient groups were assessed by the Kruskal–Wallis test. The inter-group differences were tested by the Dunn’s post-hoc test. * *p* values adjusted for age, sex and BMI (by analysis of covariance (ANCOVA)). Abbreviations: TUDCA, Tauroursodeoxycholic acid; TCA, taurocholic acid; GUDCA, glycoursodeoxycholic acid; GCA, glycocholic acid; TCDCA, taurochenodeoxycholic acid; TDCA, taurodeoxycholic acid; CA, cholic acid; UDCA, ursodeoxycholic acid; GCDCA, glycochenodeoxycholic acid; GDCA, glycodeoxycholic acid; CDCA, chenodeoxycholic acid; GLCA, glycolithocholic acid; DCA, deoxycholic acid; HDCA, hyodeoxycholic acid.

**Table 4:** Linear regression analyses—Adjusted associations between concentrations of primary and secondary bile acid concentrations and presence/absence of type 2 diabetes mellitus (T2DM) treated with or without metformin.

Linear Regression Analyses	Standardized β Coefficient(s)	*p*-Values
Log Total Primary BAs		
Adjusted model 1		
T2DM status		
Patients without T2DM (*n* = 102)	Reference	Reference
Patients with T2DM not treated with metformin (*n* = 48)	0.357	<0.0001
Patients with T2DM treated with metformin (*n* = 176)	0.279	0.005
Age (years)	0.054	0.451
Sex (men vs. women)	0.139	0.015
BMI (kg/m^2^)	0.037	0.514
Serum ALT (IU/L)	0.039	0.512
Statin use (yes vs. no)	−0.293	<0.0001
Log Total Secondary BAs		
Adjusted model 1		
T2DM status		
Patients without T2DM (*n* = 102)	Reference	Reference
Patients with T2DM not treated with metformin (*n* = 48)	0.269	0.001
Patients with T2DM treated with metformin (*n* = 176)	0.508	<0.0001
Age (years)	−0.054	0.458
Sex (men vs. women)	0.023	0.689
BMI (kg/m^2^)	−0.039	0.482
Serum ALT (IU/L)	0.017	0.775
Statin use (yes vs. no)	−0.229	0.001

Sample size, *n* = 326. Data are expressed as standardized beta coefficients as tested by univariable and multivariable linear regression analyses. Plasma levels of primary BAs and secondary BAs were logarithmically transformed before statistical analyses and were included as the dependent variable in each regression model.

**Table 5:** Linear regression analyses—Adjusted associations between plasma bile acid concentrations and presence/absence of type 2 diabetes mellitus (T2DM) treated with or without metformin.

Linear Regression Analyses	Standardized β Coefficient(s)	*p*-Values
Log TCDCA		
Adjusted model 1		
T2DM status		
Patients without T2DM (*n* = 102)	Reference	Reference
Patients with T2DM not treated with metformin (*n* = 48)	0.539	<0.0001 *
Patients with T2DM treated with metformin (*n* = 176)	0.490	<0.0001 *
Age (years)	0.033	0.618
Sex (men vs. women)	0.132	0.013
BMI (kg/m^2^)	0.018	0.722
Serum ALT (IU/L)	0.085	0.123
Statin use (yes vs. no)	−0.149	0.021
Log TDCA		
Adjusted model 1		
T2DM status		
Patients without T2DM (*n* = 102)	Reference	Reference
Patients with T2DM not treated with metformin (*n* = 48)	0.428	<0.0001 *
Patients with T2DM treated with metformin (*n* = 176)	0.449	<0.0001 *
Age (years)	−0.044	0.545
Sex (men vs. women)	0.018	0.751
BMI (kg/m^2^)	−0.060	0.291
Serum ALT (IU/L)	0.037	0.535
Statin use (yes vs. no)	−0.126	0.076
Log CA		
Adjusted model 1		
T2DM status		
Patients without T2DM (*n* = 102)	Reference	Reference
Patients with T2DM not treated with metformin (*n* = 48)	0.044	0.602
Patients with T2DM treated with metformin (*n* = 176)	−0.250	0.013
Age (years)	0.117	0.121
Sex (men vs. women)	0.010	0.869
BMI (kg/m^2^)	0.093	0.109
Serum ALT (IU/L)	−0.020	0.752
Statin use (yes vs. no)	−0.151	0.039
Log GCDCA		
Adjusted model 1		
T2DM status		
Patients without T2DM (*n* = 102)	Reference	Reference
Patients with T2DM not treated with metformin (*n* = 48)	0.432	<0.0001 *
Patients with T2DM not treated with metformin (*n* = 176)	0.50	<0.0001 *
Age (years)	0.046	0.510
Sex (men vs. women)	0.149	0.006
BMI (kg/m^2^)	0.016	0.771
Serum ALT (IU/L)	0.052	0.365
Statin use (yes vs. no)	−0.294	<0.001
Log HDCA		
Adjusted model 1		
T2DM status		
Patients without T2DM (*n* = 102)	Reference	Reference
Patients with T2DM not treated with metformin (*n* = 48)	0.018	0.821
Patients with T2DM treated with metformin (*n* = 176)	0.316	0.001 *
Age (years)	0.149	0.034
Sex (men vs. women)	−0.117	0.037
BMI (kg/m^2^)	−0.067	0.230
Serum ALT (IU/L)	−0.041	0.488
Statin use (yes vs. no)	0.012	0.859
Log GDCA		
Adjusted model 1		
T2DM status		
Patients without T2DM (*n* = 102)	Reference	Reference
Patients with T2DM not treated with metformin (*n* = 48)	0.356	<0.0001 *
Patients with T2DM treated with metformin (*n* = 176)	0.600	<0.0001 *
Age (years)	−0.067	0.343
Sex (men vs. women)	0.051	0.354
BMI (kg/m^2^)	−0.078	0.150
Serum ALT (IU/L)	0.003	0.961
Statin use (yes vs. no)	−0.172	0.011
Log GLCA		
*Adjusted model 1*		
T2DM status		
Patients without T2DM (*n* = 102)	Reference	Reference
Patients with T2DM not treated with metformin (*n* = 48)	0.135	0.109
Patients with T2DM treated with metformin (*n* = 176)	0.329	0.001 *
Age (years)	0.058	0.433
Sex (men vs. women)	−0.109	0.061
BMI (kg/m^2^)	0.019	0.733
Serum ALT (IU/L)	0.027	0.655
Statin use (yes vs. no)	−0.099	0.169
Log DCA		
*Adjusted model 1*		
T2DM status		
Patients without T2DM (*n* = 102)	Reference	Reference
Patients with T2DM not treated with metformin (*n* = 48)	0.020	0.810
Patients with T2DM treated with metformin (*n* = 176)	0.315	0.002 *
Age (years)	−0.077	0.313
Sex (men vs. women)	−0.058	0.330
BMI (kg/m^2^)	−0.003	0.961
Serum ALT (IU/L)	−0.063	0.313
Statin use (yes vs. no)	−0.071	0.332
Log TCA		
*Adjusted model 1*		
T2DM status		
Patients without T2DM (*n* = 102)	Reference	Reference
Patients with T2DM not treated with metformin (*n* = 48)	0.043	0.577
Patients with T2DM treated with metformin (*n* = 176)	−0.309	0.001 *
Age (years)	−0.021	0.762
Sex (men vs. women)	0.108	0.044
BMI (kg/m^2^)	−0.008	0.868
Serum ALT (IU/L)	0.134	0.017
Statin use (yes vs. no)	−0.160	0.016

Sample size, *n* = 326. Data are expressed as standardized beta coefficients that were tested by linear regression analysis. Each plasma BA level was logarithmically transformed before statistical analysis and was included as the dependent variable in each regression model. NB: In this table, we included only the regression models of the individual plasma BA levels that were significantly different between patients with and those without T2DM (as reported in Table 2). * Adjusted regression model 1: These associations remained statistically significant even after adjustment for multiplicity by using the Bonferroni’s correction method (*p*-value was set at 0.05/14 measured BAs = 0.0036). Abbreviations: TUDCA, tauroursodeoxycholic acid; TCA, taurocholic acid; GUDCA, glycoursodeoxycholic acid; GCA, glycocholic acid; TCDCA, taurochenodeoxycholic acid; TDCA, taurodeoxycholic acid; CA, cholic acid; UDCA, ursodeoxycholic acid; GCDCA, glycochenodeoxycholic acid; GDCA, glycodeoxycholic acid; CDCA, chenodeoxycholic acid; GLCA, glycolithocholic acid; DCA, deoxycholic acid; HDCA, hyodeoxycholic acid.

## Data Availability

All relevant data are included in the manuscript and in the Supplementary Materials.

## Supplementary Materials

The following are available online at https://www.mdpi.com/article/10.3390/metabo11070453/s1, Table S1: Plasma BA concentrations in the whole population, stratified by sex and T2DM status, Table S2: Plasma BA concentrations in the whole population, simultaneously stratified by T2DM status and use of metformin, Table S3: Plasma BA concentrations in the whole population, simultaneously stratified by T2DM status and use of incretins (i.e., DPP-IV inhibitors or GLP-1 receptor agonists), Table S4: Spearman’s correlation matrix among plasma BA concentrations, plasma lipids and fasting glucose levels.
