# Supplementary material for: Plasma Bile Acid Profile in Patients with and without Type 2 Diabetes
Source: Metabolites. 2021 Jul 14;11(7):453. doi: 10.3390/metabo11070453 (PMC8304030; doi:10.3390/metabo11070453)
Supplement: Supplementary file 1 [file metabolites-11-00453-s001.zip › metabolites-1279380-supplementary.pdf]

Supplementary Table S1 - Plasma BA concentrations in the whole population, stratified by sex and T2DM status.

	**Men (n=142)**			**Women (n=184)**		
	**Without T2DM (n=22)**	**With T2DM (n=120)**	**P-values**	**Without T2DM (n=80)**	**With T2DM (n=104)**	**P-values**
TUDCA (ng/mL)	3.5 (3.5-3.5)	3.5 (3.5-3.5)	0.676	3.5 (3.5-3.5)	3.5 (3.5-3.5)	0.583
GUDCA (ng/mL)	39.9 (16.4-96.4)	21.7 (11.9-62.1)	0.103	26.5 (12.7-55.4)	23.0 (11.1-52.7)	0.288
GCA (ng/mL)	41.8 (27.5-87.6)	50.9 (27.1-100.0)	0.664	37.9 (23.2-75.4)	38.7 (21.9-65.5)	0.540
TCDCA (ng/mL)	11.8 (7.8-31.1)	45.7 (23.9-90.3)	<0.0001	14.4 (7.8-30.9)	35.5 (20.2-61.8)	<0.0001
TDCA (ng/mL)	3.5 (3.5-11.6)	12.8 (3.5-36.6)	0.0001	3.5 (3.5-10.2)	12.8 (3.5-26.1)	0.0004
UDCA (ng/mL)	10.6 (3.5-26.5)	7.4 (3.5-26.6)	0.472	11.5 (3.5-27.9)	10.2 (3.5-26.2)	0.911
CA (ng/mL)	16.6 (6.8-42.8)	8.3 (3.5-50.7)	0.143	24.6 (9.5-100.3)	9.5 (3.5-39.9)	0.0001
GCDCA (ng/mL)	144.2 (64.2-228.7)	284.0 (145.6-565.0)	0.003	101.2 (56.9-184.0)	218.3 (124.2-357.3)	<0.0001
HDCA (ng/mL)	3.5 (3.5-3.5)	3.5 (3.5-4.9)	0.003	3.5 (3.5-3.5)	3.5 (3.5-6.8)	<0.0001
GDCA (ng/mL)	29.5 (19.8-79.9)	111.7 (53.4-234.0)	<0.0001	33.9 (16.9-72.6)	91.4 (59.4-156.4)	<0.0001
CDCA (ng/mL)	45.3 (22.9-111.2)	45.4 (14.7-143.3)	0.685	54.1 (23.2-160.7)	53.8 (14.3-121.2)	0.338
GLCA (ng/mL)	3.5 (3.5-3.5)	3.5 (3.5-5.1)	0.040	3.5 (3.5-3.5)	3.5 (3.5-8.7)	<0.0001
DCA (ng/mL)	68.4 (24.3-123.4)	123.1 (62.6-252.4)	0.020	100.8 (43.7-176.1)	151.5 (72.1-242.0)	0.016
TCA (ng/mL)	15.8 (10.6-26.8)	8.0 (5.0-19.5)	0.0002	18.6 (10.9-34.9)	7.0 (5.0-13.0)	<0.0001

Sample size, *n*=326. Data are expressed medians and interquartile ranges (in parenthesis). Differences between the patient groups were tested by the Mann-Whitney test.

Abbreviations: TUDCA, Tauroursodeoxycholic acid; TCA, taurocholic acid; GUDCA, glycoursodeoxycholic acid; GCA, glycocholic acid; TCDCA, taurochenodeoxycholic acid; TDCA, taurodeoxycholic acid; CA, cholic acid; UDCA, ursodeoxycholic acid, GCDCA, glycochenodeoxycholic acid; GDCA, glycodeoxycholic acid; CDCA, chenodeoxycholic acid; GLCA, glycolithocholic acid; DCA deoxycholic acid; HDCA, hyodeoxycholic acid.

Supplementary Table S2 - Plasma BA concentrations in the whole population, simultaneously stratified by T2DM status and use of metformin.

	**Without T2DM (n=102) (Group A)**	**With T2DM and without metformin use (n=48) (Group B)**	**With T2DM and with metformin use (n=176) (Group C)**	**P-values for trend**	**P-values for trend***	**P-values for A vs. B**	**P-values for A vs. C**	**P-values for B vs. C**
TUDCA (ng/mL)	3.5 (3.5-3.5)	3.5 (3.5-3.5)	3.5 (3.5-3.5)	0.593	0.142	0.364	0.425	0.419
GUDCA (ng/mL)	32.1 (13.7-57.7)	27.0 (12.8-79.8)	21.8 (11.1-55.2)	0.166	0.229	0.460	0.041	0.115
GCA (ng/mL)	40.4 (25.6-77.1)	62.3 (26.5-151.6)	43.4 (22.9-73.1)	0.078	0.003	0.028	0.409	0.013
TCDCA (ng/mL)	13.7 (7.9-30.9)	26.0 (68.3-170.9)	38.9 (21.7-63.4)	<0.001	<0.001	<0.001	<0.001	0.008
TDCA (ng/mL)	3.5 (3.5-10.4)	11.2 (3.5-36.6)	12.8 (3.5-28.8)	<0.001	<0.001	<0.001	<0.001	0.437
UDCA (ng/mL)	11.5 (3.5-27.4)	7.2 (3.5-26.9)	8.7 (3.5-26.4)	0.842	0.995	0.377	0.280	0.459
CA (ng/mL)	19.7 (8.6-72.4)	32.4 (5.2-158.3)	7.1 (3.5-32.5)	<0.00	<0.001	0.486	<0.001	<0.001
GCDCA (ng/mL)	109.1 (58.6-191.4)	298.1 (126.9-581.2)	239.0 (132.5-444.6)	<0.001	<0.001	<0.001	<0.001	0.194
HDCA (ng/mL)	3.5 (3.5-3.5)	3.5 (3.5-3.5)	3.5 (3.5-6.8)	<0.001	<0.001	0.019	<0.001	<0.001
GDCA (ng/mL)	31.3 (17.3-75.8)	78.8 (40.2-143.8)	109.3 (60.5-198.8)	<0.001	<0.001	<0.001	<0.001	0.048
CDCA (ng/mL)	51.7 (23.4-131.7)	71.4 (20.8-175.2)	45.6 (14.6-123.2)	0.142	0.060	0.296	0.071	0.047
GLCA (ng/mL)	3.5 (3.5-3.5)	3.5 (3.5-4.8)	3.5 (3.5-7.1)	0.003	0.009	0.023	<0.001	0.138
DCA (ng/mL)	93.1 (38.2-171.7)	72.1 (27.3-199.9)	152.4 (75.1-270.5)	<0.001	<0.001	0.329	<0.001	<0.001
TCA (ng/mL)	17.7 (10.8-32.1)	12.0 (5.8-34.5)	7.0 (5.0-12.0)	<0.001	<0.001	0.008	<0.001	<0.001

Sample size, *n*=326. Data are expressed as medians and interquartile ranges (in parenthesis). Differences among the four patient groups were tested by Kruskal-Wallis test. The inter-group differences were tested by the Dunn's post-hoc test. *P values were also adjusted for age, sex and BMI (by analysis of covariance [ANCOVA]).

Abbreviations: TUDCA, tauroursodeoxycholic acid; TCA, taurocholic acid; GUDCA, glycoursodeoxycholic acid; GCA, glycocholic acid; TCDCA, taurochenodeoxycholic acid; TDCA, taurodeoxycholic acid; CA, cholic acid; UDCA, ursodeoxycholic acid, GCDCA, glycochenodeoxycholic acid; GDCA, glycodeoxycholic acid; CDCA, chenodeoxycholic acid; GLCA, glycolithocholic acid; DCA deoxycholic acid; HDCA, hyodeoxycholic acid

Supplementary Table S3 - Plasma BA concentrations in the whole population, simultaneously stratified by T2DM status and use of incretins (i.e., DPP-IV inhibitors or GLP-1 receptor agonists).

	**Without T2DM (n=102) (Group A)**	**With T2DM and without incretins (n=130) (Group B)**	**With T2DM and with incretins (n=94) (Group C)**	**P-values for trend**	**P-values for trend***	**P-values for A vs B**	**P-values for A vs C**	**P-values for B vs C**
TUDCA (ng/mL)	3.5 (3.5-3.5)	3.5 (3.5-3.5)	3.5 (3.5-3.5)	0.977	0.529	0.304	0.316	0.498
GUDCA (ng/mL)	32.1 (13.7-57.7)	22.5 (12.3-55.8)	24.3 (11.1-61.5)	0.332	0.519	0.076	0.139	0.398
GCA (ng/mL)	40.4 (25.6-77.1)	45.5 (21.8-88.8)	45.8 (26.6-72.0)	0.922	0.682	0.359	0.368	0.498
TCDCA (ng/mL)	13.7 (7.9-30.9)	40.9 (21.9-87.0)	42.9 (21.8-64.3)	<0.001	<0.001	<0.001	<0.001	0.355
TDCA (ng/mL)	3.5 (3.5-10.4)	13.7 (3.5-36.7)	10.7 (3.5-24.7)	<0.001	<0.001	<0.001	<0.001	0.211
UDCA (ng/mL)	11.5 (3.5-27.4)	7.4 (3.5-21.3)	10.4 (3.5-29.2)	0.310	0.126	0.128	0.383	0.078
CA (ng/mL)	19.7 (8.6-72.4)	8.0 (3.5-39.0)	11.6 (3.5-52.9)	<0.001	0.006	<0.001	<0.001	0.235
GCDCA (ng/mL)	109.1 (58.6-191.4)	247.7 (135.0-478.2)	256.5 (128.8-524.6)	<0.001	<0.001	<0.001	<0.001	0.469
HDCA (ng/mL)	3.5 (3.5-3.5)	3.5 (3.5-5.9)	3.5 (3.5-5.5)	<0.001	0.002	<0.001	<0.001	0.428
GDCA (ng/mL)	31.3 (17.3-75.8)	99.8 (53.5-192.8)	93.5 (61.4-170.0)	<0.001	<0.001	<0.001	<0.001	0.349
CDCA (ng/mL)	51.7 (23.4-131.7)	40.8 (13.1-111.8)	54.4 (24.2-162-6)	0.105	0.038	0.041	0.442	0.032
GLCA (ng/mL)	3.5 (3.5-3.5)	3.5 (3.5-5.6)	3.5 (3.5-7.0)	0.003	0.034	<0.001	<0.001	0.098
DCA (ng/mL)	93.1 (38.2-171.7)	116.8 (59.1-219.1)	163.2 (70.1-298.7)	0.002	0.005	0.002	<0.001	0.042
TCA (ng/mL)	17.7 (10.8-32.1)	7.0 (5.0-18.0)	7.0 (5.0-10.5)	<0.001	<0.001	<0.001	<0.001	0.150

Sample size, *n*=326. Data are expressed medians and interquartile ranges (in parenthesis). Differences among the four groups of patients were tested by Kruskal-Wallis test. The inter-group differences were tested by the Dunn's post-hoc test. *P values were also adjusted for age, sex and BMI (using analysis of covariance [ANCOVA]).

Abbreviations: TUDCA, Tauroursodeoxycholic acid; TCA, taurocholic acid; GUDCA, glycoursodeoxycholic acid; GCA, glycocholic acid; TCDCA, taurochenodeoxycholic acid; TDCA, taurodeoxycholic acid; CA, cholic acid; UDCA, ursodeoxycholic acid, GCDCA, glycochenodeoxycholic acid; GDCA, glycodeoxycholic acid; CDCA, chenodeoxycholic acid; GLCA, glycolithocholic acid; DCA deoxycholic acid; HDCA, hyodeoxycholic acid

Supplementary Table S4 – Spearman’s correlation matrix among plasma BA concentrations, plasma lipids and fasting glucose levels.

	TUDCA (ng/mL)	GUDCA (ng/mL)	GCA (ng/mL)	TCDCA (ng/mL)	TDCA (ng/mL)	UDCA (ng/mL)	CA (ng/mL)	GCDCA (ng/mL)	HDCA (ng/mL)	GDCA (ng/mL)	CDCA (ng/mL)	GLCA (ng/mL)	DCA (ng/mL)	TCA (ng/mL)	Total cholesterol (mmol/L)	LDL-C (mmol/L)	HDL-C (mmol/L)	TG (mmol/L)	Glucose (mg/dL)
**TUDCA (ng/mL)**	1.00																		
**GUDCA (ng/mL)**	0.332*	1.00																	
**GCA (ng/mL)**	0.312*	0.539*	1.00																
**TCDCA (ng/mL)**	0.291*	0.316*	0.661*	1.00															
**TDCA (ng/mL)**	0.227*	0.134*	0.510*	0.667*	1.00														
**UDCA (ng/mL)**	0.213*	0.713*	0.194*	0.060	-0.124*	1.00													
**CA (ng/mL)**	0.126*	0.272*	0.262*	0.076	-0.093	0.411*	1.00												
**GCDCA (ng/mL)**	0.278*	0.579*	0.737*	0.794*	0.478*	0.269*	0.161*	1.00											
**HDCA (ng/mL)**	-0.061	-0.110	-0.051	0.046	0.213*	-0.082	-0.205*	0.040	1.00										
**GDCA (ng/mL)**	0.197*	0.302*	0.498*	0.493*	0.788*	0.065	-0.073	0.602*	0.330*	1.00									
**CDCA (ng/mL)**	0.166*	0.411*	0.322*	0.249*	0.001	0.636*	0.771*	0.383*	-0.143*	0.087	1.00								
**GLCA (ng/mL)**	0.099	0.232*	0.245*	0.283*	0.364*	0.144*	0.067	0.389*	0.259*	0.496*	0.172	1.00							
**DCA (ng/mL)**	0.007	0.130*	0.098	0.023	0.338*	0.386*	0.252*	0.142*	0.298*	0.588*	0.449*	0.351*	1.00						
**TCA (ng/mL)**	0.281*	0.279*	0.692*	0.456*	0.396*	0.020	0.282*	0.293*	-0.238*	0.085	0.191*	0.040	-0.141*	1.00					
**Total cholesterol (mmol/L)**	0.034	0.036	0.019	-0.251*	-0.131*	0.020	0.210*	-0.183*	-0.192*	-0.183*	0.046	-0.181*	-0.072	0.243	1.00				
**LDL-C (mmol/L)**	0.035	0.021	0.011	-0.271*	-0.136*	0.031	0.239*	-0.215*	-0.226*	-0.212*	0.055	-0.217*	-0.078	0.260*	0.935*	1.00			
**HDL-C (mmol/L)**	-0.0154	-0.045	-0.033	-0.056	0.011	-0.119*	-0.121*	-0.057	0.110	0.052	-0.125*	0.052	-0.013	-0.037	0.218*	0.008	1.00		
**TG (mmol/L)**	-0.042	0.051	0.023	0.075	0.027	0.144*	0.013	0.081	0.015	0.039	0.073	0.007	0.095	-0.038	0.057	0.012	-0.489*	1.00	
**Glucose (mg/dL)**	-0.026	-0.038	0.036	0.327*	0.264*	-0.007	-0.175*	0.256*	0.253*	0.333*	0.010	0.237*	0.186*	-0.245*	-0.346*	-0.357*	-0.026	0.188*	1.00

Data are expressed as Spearman's rank correlation coefficients (rho coefficients). *p<0.05

Abbreviations: TUDCA, tauroursodeoxycholic acid; TCA, taurocholic acid; GUDCA, glycoursodeoxycholic acid; GCA, glycocholic acid; TCDCA, taurochenodeoxycholic acid; TDCA, taurodeoxycholic acid; CA, cholic acid; UDCA, ursodeoxycholic acid, GCDCA, glycochenodeoxycholic acid; GDCA, glycodeoxycholic acid; CDCA, chenodeoxycholic acid; GLCA, glycolithocholic acid; DCA deoxycholic acid; HDCA, hyodeoxycholic acid; TG, triglycerides.
